# Deep-Learning-Based Automated Identification and Visualization of Oral Cancer in Optical Coherence Tomography Images

**DOI:** 10.3390/biomedicines11030802

**Published:** 2023-03-06

**Authors:** Zihan Yang, Hongming Pan, Jianwei Shang, Jun Zhang, Yanmei Liang

**Affiliations:** 1 Institute of Modern Optics, Tianjin Key Laboratory of Micro-Scale Optical Information Science and Technology, Nankai University, Tianjin 300350, China; zhyang@mail.nankai.edu.cn (Z.Y.); phm19960428@163.com (H.P.); 2 Department of Oral Pathology, Tianjin Stomatological Hospital, Hospital of Stomatology, Nankai University, Tianjin 300041, China; 15102232467@163.com; 3 Department of Oral-Maxillofacial Surgery, Tianjin Stomatological Hospital, Hospital of Stomatology, Nankai University, Tianjin 300041, China; zjsurgeon@126.com

**Keywords:** optical coherence tomography, oral cancer, identification, deep learning, machine learning

## Abstract

Early detection and diagnosis of oral cancer are critical for a better prognosis, but accurate and automatic identification is difficult using the available technologies. Optical coherence tomography (OCT) can be used as diagnostic aid due to the advantages of high resolution and non-invasion. We aim to evaluate deep-learning-based algorithms for OCT images to assist clinicians in oral cancer screening and diagnosis. An OCT data set was first established, including normal mucosa, precancerous lesion, and oral squamous cell carcinoma. Then, three kinds of convolutional neural networks (CNNs) were trained and evaluated by using four metrics (accuracy, precision, sensitivity, and specificity). Moreover, the CNN-based methods were compared against machine learning approaches through the same dataset. The results show the performance of CNNs, with a classification accuracy of up to 96.76%, is better than the machine-learning-based method with an accuracy of 92.52%. Moreover, visualization of lesions in OCT images was performed and the rationality and interpretability of the model for distinguishing different oral tissues were evaluated. It is proved that the automatic identification algorithm of OCT images based on deep learning has the potential to provide decision support for the effective screening and diagnosis of oral cancer.

## 1. Introduction

Oral cancer is one of the most common cancers in the head and neck [1]. In terms of the pathogenesis of oral cancer, the predominant type of oral cancer is oral squamous cell carcinoma (OSCC) with a long preclinical stage [2]. In addition, precancerous lesions (oral potentially malignant disorder), such as homogeneous leukoplakia and nonhomogeneous leukoplakia, are at risk of malignant transformation [3]. Despite the advancement in targeted cancer therapy, survival rates for oral cancer have remained flat over the last 50 years [4]. Fortunately, the patient’s survival can be improved if the OSCC can be detected and diagnosed early for appropriate treatments [5]. The study indicated that the 5-year survival rate can increase from less 30% to 83% with early detection [6]. Therefore, it is critical that oral cancer can be diagnosed and treated in the pre- or early cancerous stages.

The conventional visual examination is the most commonly screening procedure for oral lesions, but its sensitivity and specificity vary greatly [7]. Auxiliary methods, such as, toluidine blue, auto-fluorescence, or non-linear microscopy have been studied [8,9,10,11,12]. However, there are some limitations, such as the safety assessment of chemiluminescence methods, the lack of three-dimensional (3D) information of fluorescence, or the limited field of view and depth of microscopic methods. While histopathology is still the gold standard, this processing is invasive and time-consuming.

The study has shown that the thickness of oral mucosa (epithelium and lamina propria) is less than 1 mm [13]. For oral cavity imaging with microscopic techniques, their penetration depth is limited, which may not be deep enough to investigate the existence of basement membrane. In contrast, optical coherence tomography (OCT) has the advantages of high-resolution (1–20 μm), real-time and large-depth (1–2 mm) imaging which is suitable for imaging oral mucosa. OCT has been applied in biomedical fields since it was first introduced in 1991 [14], such as ophthalmology [15], cardiology [16], gastroenterology [17], and dermatology [18]. In the oral cavity, studies based on OCT have been attempted to differentiate benign and OSCC by different structural or optical indicators, including the thickness of the epithelium, the intactness of basement membrane, or optical scattering properties [19,20,21]. It has been proved that OCT can enable imaging of oral mucosa and identification of the morphological structures.

Automatic image recognition and classification play an important role in biomedicine. To identify oral lesions automatically, texture feature-based methods were proposed. Krishnan et al. made use of high-order spectra, local binary pattern and laws texture energy from histopathological images to identify oral sub-mucous fibrosis [22]. Thomas et al. used the grey level co-occurrence matrix and grey level run-length for classification of oral cancer in digital camera images [23]. Recently, our laboratory has studied the use of texture features to distinguish salivary gland tumors [24], as well as OSCC [25] in OCT images.

In addition, deep learning has been surprisingly successful in recent years [26,27,28]. In the field of biomedicine, deep learning has been developed for disease classification, object segmentation and image enhancement. Aubreville et al. presented and evaluated an automatic approach for OSCC diagnosis using deep learning on confocal laser endomicroscopy images [29]. Welikala et al. assessed two deep-learning-based computer vision approaches for the automated detection and classification of oral lesions in photographs [30]. However, there is no research on deep-learning-based automatic recognition of oral cancer in OCT images. 

The goal of this study is to explore the potential of automatic recognition of oral cancer based on deep learning in OCT images and evaluate the effectiveness by identifying precancerous and cancerous tissues. In addition, feature visualization is also studied to evaluate the rationality and interpretability of the network. It has the great potential to assist clinicians in screening and diagnosis of oral cancer and precancerous lesions.

## 2. Materials and Methods

### 2.1. Sample Preparation and Data Acquisition

Fresh tissue samples investigated in this study were obtained from the Tianjin Stomatology Hospital, China. All procedures performed in this study were in accordance with the ethical standards of the Ethics Committee of Tianjin Stomatological Hospital. These samples came from 19 patients who were diagnosed with oral diseases, including leukoplakia with hyperplasia (LEH) and OSCC. The normal and diseased oral tissues were sequentially scanned, and then were fixed and stained with H&E. The slices were evaluated by an experienced pathologist. The details about the OCT system and imaging protocol were described in the previous work [31,32].

### 2.2. Establishment of the Data Set

**Figure 1:**
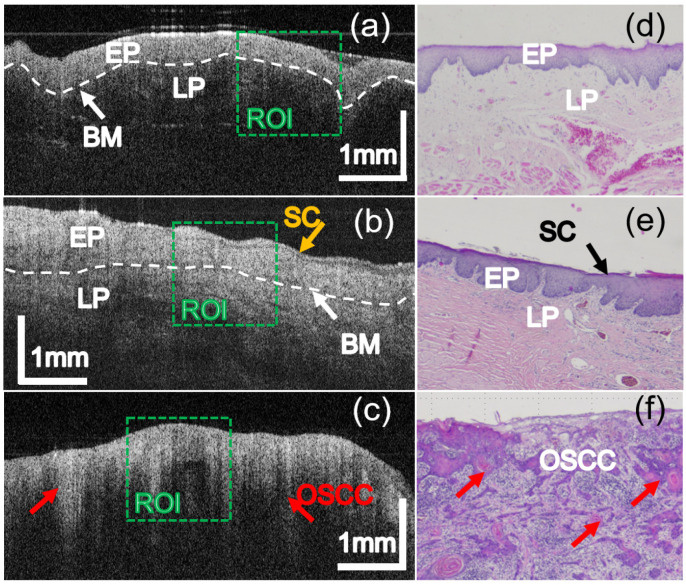
Morphological characteristics and statistical analysis of oral tissues. The representative OCT images of normal mucosa (**a**), LEH (**b**), and OSCC (**c**) and corresponding histopathological images (**d**–**f**). The ROI indicates 256 × 256 pixels.

Different morphological features of oral tissues were marked in Figure 1. Figure 1a,d show the OCT image and the corresponding histopathologic image of normal mucosa. It can be found that the epithelium (EP) and the lamina propria (LP) are clearly distinguishable due to the different optical scattering intensity, which corresponds well to the histopathological image (Figure 1d). The boundary of EP and LP is called the basement membrane (BM), as shown by the white dashed curve. The typical OCT image and the corresponding histopathologic image of LEH are shown in Figure 1b,e. We can see a boundary (BM) similar to that of normal mucosa from Figure 1b,e. It is worth noting that the thickness of EP is increased and the stratum corneum (SC) can also be observed. In contrast, the epithelial cells of OSCC proliferate maliciously, resulting in the destruction of BM. Moreover, due to the aggregation of cancer cells, the distribution of optical scattering signal appears as cord-like in the OCT image, as indicated by the red arrows in Figure 1c,f.

According to the above analysis of morphological characteristics of different oral tissues in OCT images, OCT images matched with the histopathological images were manually segmented to the appropriate size (256 × 256 pixels) as the regions of interest (ROIs), which contain information unique to different tissues, as shown in the green square boxes in Figure 1. After the segmentation, a total of 13,799 OCT images of ROIs were used to establish the data set. In order to avoid data bias, we randomly selected OCT images from some patients for training and others for test, as described in detail in Table 1. 

### 2.3. CNN Architecture

Three CNNs, including LeNet-5, VGG16, and ResNet18, were used for the classification and identification of these oral tissues (Figure S1). As one of the most basic and earliest proposed deep learning networks, LeNet-5 has a simple network structure and a small number of parameters [33]. There are two convolution layers and three fully connected layers in LeNet-5. The rectified linear unit (ReLU) activation function and max pooling operation are used after each convolutional layer. VGG16 is composed of 13 convolution layers and 3 full connection layers, in which ReLU is used as activation functions after every two convolutional layer and full connected layer [34]. VGG16 changed the convolution mode, set multiple convolution kernels, increased the channel, and reduced the matrix width and height through pooling. With the deepening of layers of VGG16, the amount of computation increases. ResNet18 consists of 18 layers with weights, including the convolutional layers and the fully connected layers. ResNet18 avoided the vanishing gradient and reduced computation amount by skip connections [35].

### 2.4. Training and Classification

**Figure 2:**
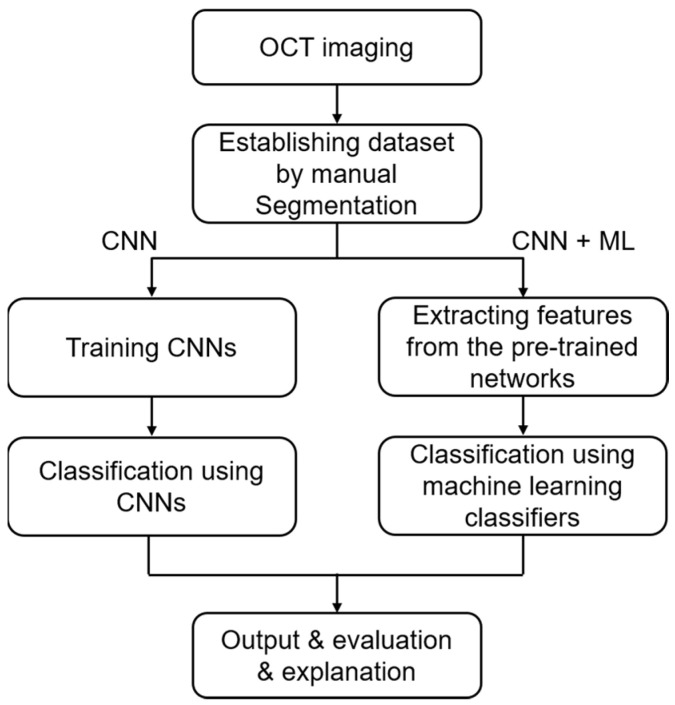
Flowchart of the oral tissue classification experiment.

Figure 2 is the flowchart of our experiment. CNNs were firstly trained by using random initialization parameters. To reduce the risk of overfitting, 10-fold cross-validation was performed. Then, the independent test set (never seen by the network before) was used to test the classification performance of different CNNs. In this study, the CNNs were implemented under the PyTorch framework. The batch size is set to 32, the cross-entropy is used as the loss function, and Adam is used as the optimizer with a learning rate of 0.0001, momentum of 0.9.

Further, considering that CNN can perform feature extraction, we used CNN as the feature extractor and machine learning (ML) as the classifier to evaluate the effectiveness in oral tissues classification (CNN + ML). Here, the features were extracted from the last layer before the classification layer (the last fully connected layer) of the pre-trained networks. After that, the feature dimensionality reduction was carried out by the principal component analysis (PCA) algorithm [25]. Finally, three kinds of ML classifiers were used, including decision tree (DT), random forest (RF), and support vector machine (SVM) [25]. 

In this method, transfer learning was applied and the networks were trained on the ImageNet dataset in advance. Transfer learning can transfer the acquired powerful skills to relevant problems, thus saving time and computing costs [36]. For the classifiers we used, DT model is a kind of tree structure, which is composed of a series of nodes, and each node represents a feature. RF is an algorithm that integrates multiple decision trees through ensemble learning. The random vector is used to generate the ensemble of trees and control the growth of each tree in the ensemble, which can significantly improve the classification accuracy. The number and the depth of tree nodes were used to optimize the best results. Multi-class SVM classifiers with Gaussian radial basis function as the kernel function were employed and the non-linear decision boundary was obtained. The penalty factors C and gamma were optimized for SVM.

In order to apply this method to a common scenario, the algorithms were executed on a desktop computer with an eight-core Intel Xeon 3.5 GHz (E5-1620) processor and a 24 GB random-access memory using the Python programming software (Version 3.7.3).

### 2.5. Evaluation Indicators

To evaluate the performance of different CNNs and approaches in distinguishing oral tissues, four metrics including sensitivity (*Sen*), specificity (*Spe*), precision (*Pre*), and accuracy (*Acc*) were calculated.

(1) $$Sen=\frac{TP}{TP+FN}$$

(2) $$Spe=\frac{TN}{TN+FP}$$

(3) $$Pre=\frac{TP}{TP+FP}$$

(4) $$Acc=\frac{TP+TN}{TP+FP+TN+FN}$$

*TP*: true positives, *FP*: false positives, *FN*: false negatives, *TN*: true negatives

In addition, receiver operating characteristic curves (ROCs) were plotted and areas under ROC (AUCs) were also calculated. ROCs and AUCs can be used to describe the classification performance of models objectively. The degree of convergence of the networks was determined by the loss value obtained by the loss function (cross entropy loss).

### 2.6. Visualization

To more directly display classification performance, the predictions of the CNNs were calculated. We extracted 384 overlapping patches from each image. Each patch was input the trained network one by one, and the prediction results were visualized using pseudo-color map.

To enhance interpretability of networks, gradient weighted class activation mapping (Grad-CAM) technique was used to highlight the important regions in the OCT images of oral tissues, which creates the visual explanation for CNNs and helps determine more information about the models when performing detection or prediction work [37].

## 3. Results

### 3.1. Identification Using CNN Alone

All the CNNs were trained and tested using PyTorch, which is a deep learning framework enabling fast implementation. After 40 epochs of training, loss values of three kinds of CNNs tend to converge (Figure S2). The results of three CNNs using 10-fold cross-validation were shown in Table S1 and the accuracy of the CNN models was verified.

**Figure 3:**
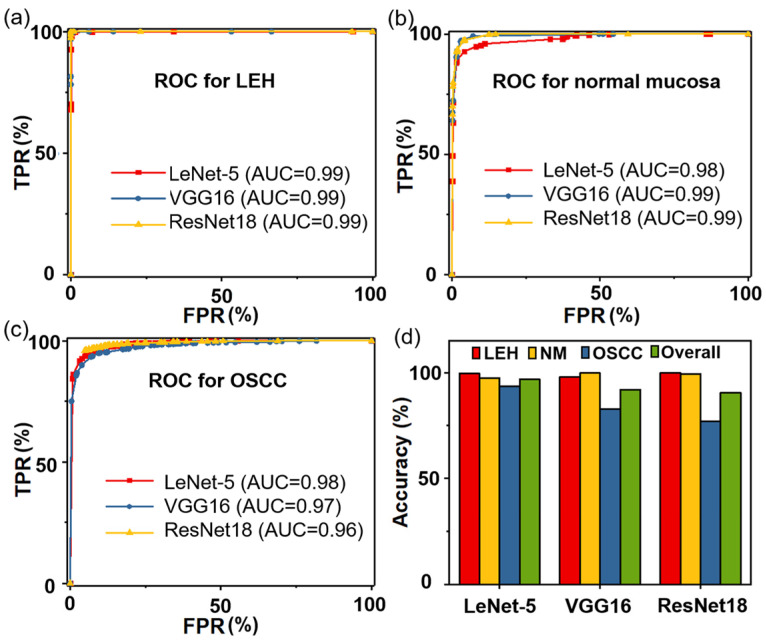
The classification evaluation of three kinds of CNNs. (**a**–**c**) are the ROCs and AUCs of distinguishing three types of tissues using these three CNNs, respectively. (**d**) is the classification accuracies of three types of tissues with three types of CNNs. TPR: true positive rate, FPR: false positive rate.

The performances of three kinds of CNNs were presented in Figure 3a–c, respectively. It is observed that three kinds of CNNs are capable of distinguishing each type of tissue, especially for LEH (AUC = 0.99 for all CNNs). The classification accuracies were further calculated, as shown in Figure 3d. For LeNet-5, the classification accuracies of LEH, normal mucosa, and OSCC are 99.56%, 97.51%, and 93.37%, respectively. For VGG16, the accuracy of each type of tissue is 97.87%, 99.77%, and 82.79%, respectively. For ResNet18, the accuracy of each class is 99.87%, 99.32%, and 77.01%, respectively. The overall accuracies of using LeNet-5, VGG16, and ResNet18 are 96.76%, 91.94%, and 90.43%, respectively. 

### 3.2. Identification Using CNN + ML

**Figure 4:**
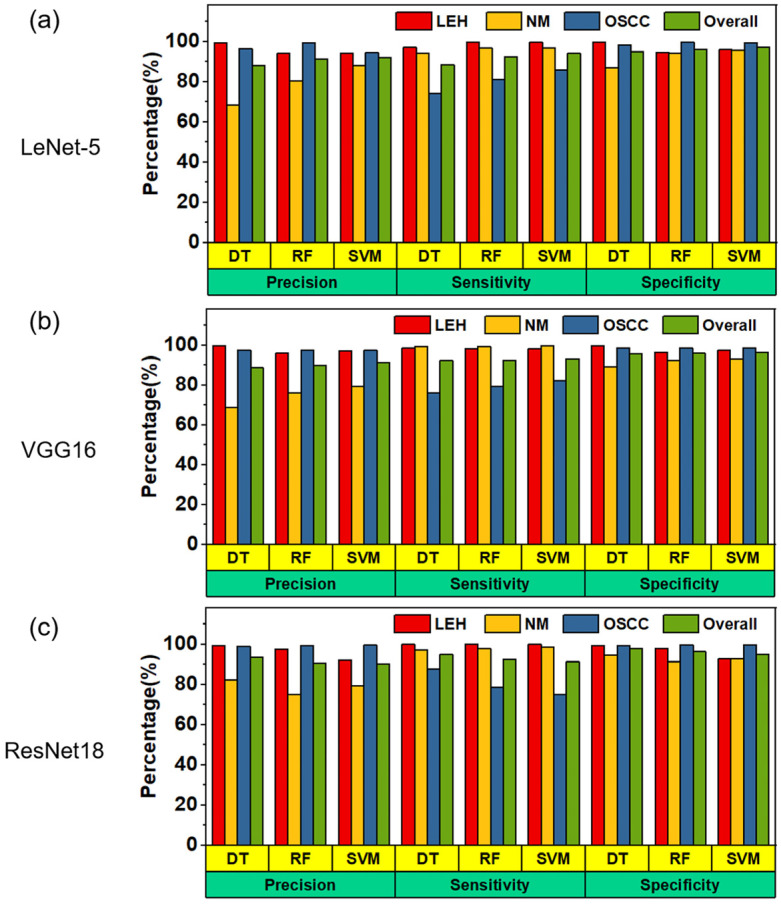
The performance comparison of the three classifiers (DT, RF, and SVM) when using LeNet-5 (**a**), VGG16 (**b**), and ResNet18 (**c**) as a feature extractor, respectively.

Given that ML-based methods often require manual feature extraction and feature selection, it brings about contingency and inconvenience for accurate recognition. To address these issues, we used CNNs as feature extractor, and then used ML as classifier to identify different tissues in OCT images. Figure 4 shows the performance of three classifiers after feature extraction using different networks. For LeNet-5 as feature extractor, the overall accuracies of SVM, DT, and RF are 92.52%, 88.23%, and 91.53%, respectively. For VGG16 as feature extractor, the overall accuracies of SVM, DT, and RF are 91.33%, 89.42%, and 90.52%, respectively. For ResNet18 as feature extractor, the overall accuracies of SVM, DT, and RF are 89.51%, 90.12%, and 91.01%, respectively. The corresponding ROC curves show that CNNs combined with SVM can obtain the best results (Figure S3).

**Figure 5:**
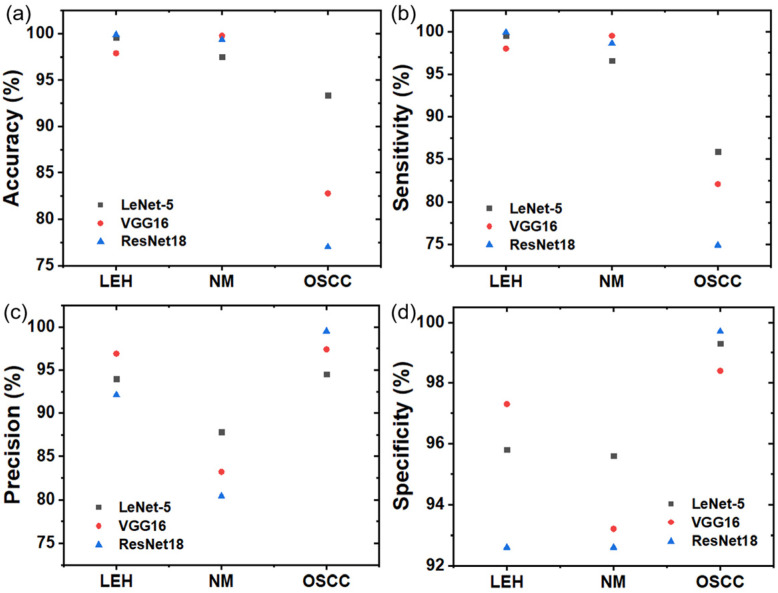
The performance comparison of using SVM as classifier and CNNs as feature extractor. Accuracy (**a**), sensitivity (**b**), precision (**c**), and specificity (**d**) of classification models using LeNet-5, VGG16, and ResNet18 as feature extractor, respectively.

To further evaluate the best strategy, the comparison of using CNNs as feature extraction and SVM as classifier is shown in Figure 5. As a whole, it can be found that SVM combined with LeNet-5 achieved best results, whose overall classification accuracy is 92.52%. Accordingly, the precision, sensitivity, and specificity of identifying normal mucosa, LEH, and OSCC are shown in Table 2. 

### 3.3. Performance Evaluation of Two Strategies

Two classification strategies including the use of CNN alone and the use of CNN combined with ML were evaluated from accuracy (Table 3). It can be found that if only CNN is used, LeNet-5 obtained the highest accuracy of 96.76%; if CNN + ML was used, LeNet-5 combined with SVM achieved the highest accuracy of 92.52%. Therefore, the evaluation between two best strategies was implemented. The confusion matrices were shown in Figure S4. In addition, the statistics analysis of two best strategies was performed (Figure S5). Based on the two-sample student’s *t* test, there was a statistical significance between the accuracies of LeNet-5 (CNN only) and LeNet-5 combined with SVM (CNN + ML) at *p* < 0.05.

In addition, the training time spent on both strategies was also assessed (Table 4). For using CNN alone, due to the difference in the number of network structure and parameters, the average time of each epoch of training LeNet-5 is much less than that of training VGG16, and ResNet18. Similarly, LeNet-5 need the least time for the network to converge. For CNN + ML, it took less time to train ML classifiers, although it required extracting features from the CNNs.

### 3.4. Predictive Visualization

**Figure 6:**
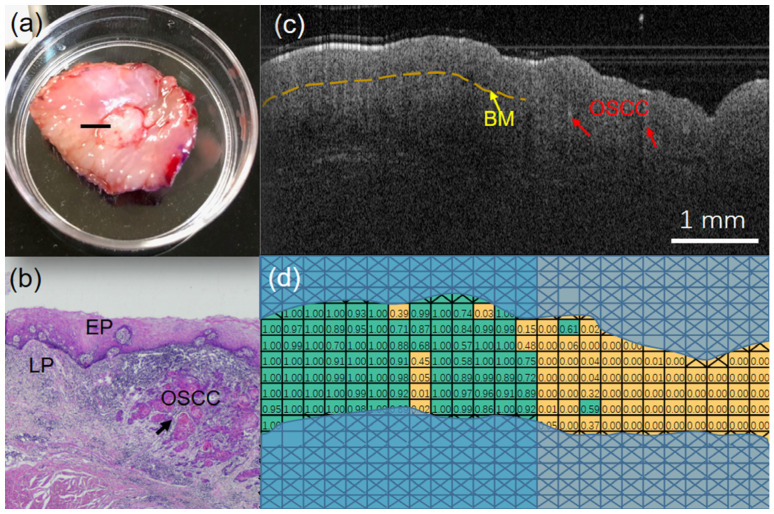
OCT imaging and prediction results at the junction between normal mucosa and OSCC. (**a**) is a photograph of the excised tissue. (**b**) is the corresponding histopathological image with the normal region on the left and the cancerous region on the right. (**c**) is the OCT image at the black line of (**a**). (**d**) is the corresponding prediction visualization.

Figure 6 shows the predictive visualization results at the junction between normal mucosa and OSCC using a trained CNN model. Figure 6a shows the imaging area in the photograph of the excised sample. According to the histopathological image (Figure 6b), the normal area and the cancerous area are located on the left and the right sides of the image, respectively. From the corresponding OCT image, as shown in Figure 6c, there is a slight distinction between normal and cancerous regions. We can see that there is a slight BM structure on the left, but not on the right. After the patches are input into the network, the differences between the left and the right of the predicted results can be clearly seen in Figure 6d and are consistent with the histopathological image.

### 3.5. Grad-CAM Visualization

**Figure 7:**
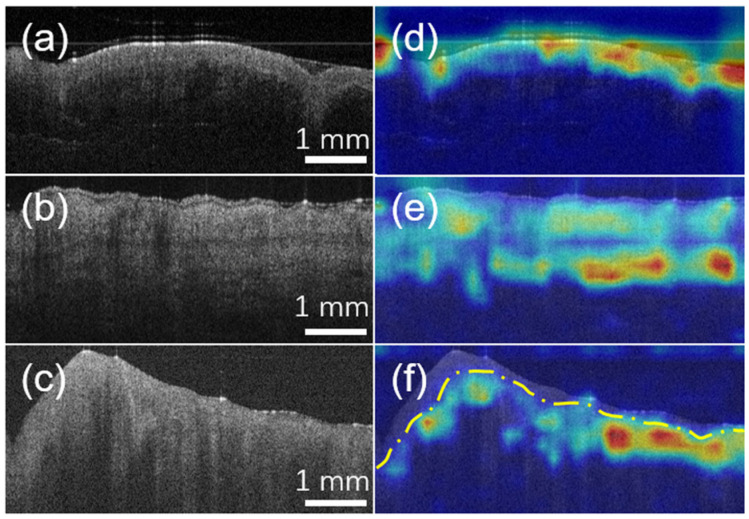
Visualization on OCT images of oral tissues using Grad-CAM. (**a**–**c**) are representative OCT images of normal mucosa, LEH, and OSCC, respectively. (**d**–**f**) are the corresponding activation maps with unique feature aggregations, respectively.

The interpretability of neural networks using Grad-CAM was also evaluated for making efficient and confident decisions. As shown in Figure 7, different oral tissues showed different characteristics in a trained CNN model. Figure 7a is an OCT image of normal mucosa and the corresponding activation map is shown in Figure 7d. It can be found that the network primarily extracts the EP. The OCT image and corresponding activation map of LEH are shown in Figure 7b,e. It can be found that the thickened EP and LP are highlighted. The OCT image of OSCC is shown in Figure 7c. The cord-like morphological structures can be seen in the OCT image due to the accumulation of cancer nests. From Figure 7f, the highlighted area is mainly the aggregation area of cancer cells, and the neural network pays more attention to this area (the area below indicated by the yellow dotted line). These results are consistent with histological findings as described above. It demonstrated that the network learned different characteristics of oral lesions to distinguish each type of oral tissues.

## 4. Discussion

We studied deep-learning-based identification of oral precancerous and cancerous lesions in this paper. Firstly, three basic kinds of CNNs were trained and evaluated based on oral OCT image data sets that includes normal mucosa, LEH, and OSCC. Next, to avoid the contingency and inconvenience of traditional machine learning methods when extracting features manually, CNNs were used as the feature extractors. DT, RF, and SVM were trained by using the activations of the last layer before the classification layer. Both of two strategies obtained excellent classification results. In addition, the performance of the networks was further verified by feature visualization in OCT images.

Compared to traditional ML methods, deep learning reduces the dependence on feature extraction. The methods used in deep learning are substantially effective to describe the characteristics of images than texture features. Through comparison on the same dataset, it can be found that using CNN alone especially using LeNet-5 obtained better classification results than that of using CNN + ML, whereas the training time of the former about several hundreds of times longer than that of the latter.

To speed up network training, and expand the data set, we segmented the entire image into a certain size. The images were split according to the following segmentation criteria:

(1) According to the requirements of the network on the input size, the size of ROI is determined as 256 × 256 pixels, which can speed up network training compared to the whole image being input.
(2) Each ROI must contain the unique characteristics of oral tissue, for example, the epithelium and lamina propria must be included for normal tissue. It can be found that the size of 256 × 256 pixels can not only include the features of oral tissue, but also effectively reduce the interference of background area.
(3) In order to effectively use the information in the image, we selected ROI areas in an overlapping approach.
(4) Areas with poor image quality were discarded, such as areas without focus due to large fluctuation of tissue surface.

Similar to the data augmentation used in conventional deep learning, the ROIs we obtained were used to make the network learn invariable features and prevent the network from learning irrelevant features, thus improving network performance. 

According to the high-performance model, we evaluated the oral tissue OCT images to automatically predict and visualize the lesions, which will be more in line with the actual needs and suitable for intuitive judgement between normal and cancerous areas. 

In addition, neural networks are often seen as black boxes in disease screening because they provide only the final diagnosis of the subject without any details of the basis of the diagnosis, which brings a major challenge to the application of artificial intelligence in clinical devices. In our study, the Grad-CAM was used to visualize the important regions in the oral tissue OCT images. It can be found that three types of oral tissues showed different characteristics in the deep learning network. Moreover, the aggregation of characteristics can reflect the unique feature of each oral tissue. Through the feature visualization, there is reasonable basis for understanding the model classification and identification.

Although microscopic or histopathological examination of tissue is the gold standard, an accurate result of biopsy may depend on the clinician’s experience and confidence, and the selection of biopsy site. A more accurate diagnosis was achieved via multiple site biopsies and larger volume samples [38], which seems to be more important but makes patients more painful for oral precancerous lesions suspected to be malignant transformation. 

In addition, intraoperative frozen section biopsy for surgical margin is a routine procedure after oral cancers are resected en bloc. Surgical margins are usually selected according to surgeons’ estimate for suspicious sites of inadequate resection, which may result in omission of positive margins. 

As auxiliary tools, imaging techniques have become indispensable in clinic, where image identification algorithms play an important role [39]. This study extends our prior work in oral cancer, which demonstrated the feasibility of OCT image-based identification of OSCC and normal mucosa by using optical parameters as markers to establish the optical attenuation model and using texture-based ML models [21,25]. 

It is noted that the robustness of deep-learning-based identification methods is worth further exploration with different OCT systems. Deep-learning-based identification is performed by extracting differential features from OCT images of oral tissues. That is, the OCT images of different lesions contain differentiated morphological features, which are recorded by the OCT system. In this case, if different OCT systems are employed, such as different wavelengths or different bandwidths, then the acquired OCT images contain different features. Therefore, collaboration between different devices to obtain more data and conduct robust research based on deep learning is the next direction.

Fortunately, using the powerful learning capabilities of deep learning and the advantage of high-speed and high-resolution imaging of swept-source OCT system, it laid a foundation for guiding clinicians to screen and resect tumors in real time accurately. 

## 5. Conclusions

In conclusion, the feasibility and validity of automatic recognition strategies for OSCC based on OCT and deep learning have been demonstrated. The interpretability of disease assessment was further investigated by visualizing network feature maps. It is proved that automatic identification methods combining the powerful learning capabilities of deep learning with the advantages of OCT imaging are feasible, which is expected to provide decision support for effective screening and diagnosis of oral cancer and precancerous tissues.

## Figures and Tables

**Table 1:** Information of the patients and the partitioning of the data set.

Dataset	Normal *	LEH	OSCC	Total
Patients’ number	-	5	14	19
Age (median [range])	-	62 (37–73)	60 (29–69)	
Gender (male/female)	-	3/2	7/7	10/9
Training set				
Patients’ number	-	3	10	13
OCT images	2151	3639	3947	9737
Test set				
Patients’ number	-	2	4	6
OCT images	1043	1601	1418	4062

* OCT images of normal mucosa were captured from the normal part of the abnormally excised tissues. The normal area was determined to be at least 1 cm away from abnormal area under the guidance of an experienced surgeon.

**Table 2:** The precision, sensitivity, and specificity of identifying NM, LEH, and OSCC using SVM combined with LeNet-5.

Parameter	NM	LEH	OSCC
Precision (%)	87.8	94.0	94.5
Sensitivity (%)	90.7	99.5	86.0
Specificity (%)	95.6	95.8	97.3

**Table 3:** Overall accuracy (%) of the two classification strategies.

Model	Classifier	LeNet-5	VGG16	ResNet18
CNN alone	-	96.76	91.94	90.43
CNN + ML	DT	87.23	89.42	90.51
	RF	91.53	90.52	90.01
	SVM	92.52	91.33	89.51

**Table 4:** Time of training CNNs and machine learning classifiers.

Model	LeNet-5	VGG16	ResNet18
CNN alone			
Each epoch/s	228	2891	1618
Convergence/s	9120	115,640	64,720
CNN + ML			
Feature extraction/s	86	710	481
DT/s	0.57	7.12	0.88
RF/s	0.27	1.25	0.29
SVM/s	15	22	1.56

## Data Availability

Data will be available from the corresponding author upon reasonable request.

## Supplementary Materials

The following supporting information can be downloaded at: https://www.mdpi.com/article/10.3390/biomedicines11030802/s1, Table S1: The accuracy (%) of identifying oral tissues using 10-fold cross-validation of different CNN models; Figure S1: Schematic architectures of three typical CNNs; Figure S2: The training loss curves of three CNN models; Figure S3: The ROC curves for SVM, DT, and RF as classifiers and LeNet-5, VGG16, and ResNet18 as feature extractors; Figure S4: Confusion matrices of two strategies; Figure S5: Statistical analysis of two strategies based on student’s *t* test.
