# Supplementary material for: Deep-Learning-Based Automated Identification and Visualization of Oral Cancer in Optical Coherence Tomography Images
Source: Biomedicines. 2023 Mar 6;11(3):802. doi: 10.3390/biomedicines11030802 (PMC10044902; doi:10.3390/biomedicines11030802)
Supplement: Supplementary file 1 [file biomedicines-11-00802-s001.zip › biomedicines-2199719-supplementary.pdf]

Deep-Learning-Based Automated Identification and Visualization of Oral Cancer in Optical Coherence Tomography Images

Captions of table and figures:

Table S1: The accuracy (%) of identifying oral tissues using 10-fold cross-validation of different CNN models.

Figure S1: Schematic architectures of three typical CNNs. As two examples of the parameter annotations, "5×5 conv, 6" represents a convolutional layer with 6 filters and the filter size of 5×5, and "FC 120" represents a fully connected layer with 120 neurons.

Figure S2: The training loss curves of three CNN models.

Figure S3: The ROC curves for SVM, DT, and RF as classifiers and LeNet-5, VGG16, and ResNet18 as feature extractors.

Figure S4: Confusion matrices of two strategies. (a) LeNet-5. (b) LeNet-5 with SVM as classifier and LeNet-5 as feature extractor.

Figure S5: Statistical analysis of two strategies based on student's *t* test.

Table S1: The accuracy (%) of identifying oral tissues using 10-fold cross-validation of different CNN models.

Network	Fold index										
	1	2	3	4	5	6	7	8	9	10	Avg
LeNet-5	95.52	94.08	95.56	97.17	94.85	96.93	95.62	97.52	98.83	95.84	96.19
VGG16	91.64	92.58	92.55	92.63	93.07	93.24	92.76	92.05	91.14	89.49	92.12
ResNet18	93.86	91.84	88.75	90.49	89.53	91.24	89.66	90.24	90.46	91.63	90.77

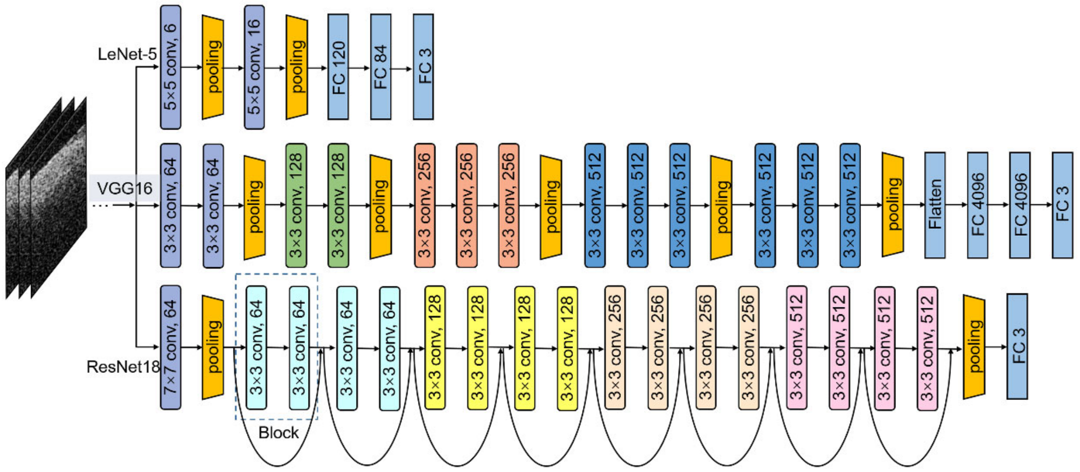

Figure S1: Schematic architectures of three typical CNNs. As two examples of the parameter annotations, “5×5 conv, 6” represents a convolutional layer with 6 filters and the filter size of 5×5, and “FC 120” represents a fully connected layer with 120 neurons.

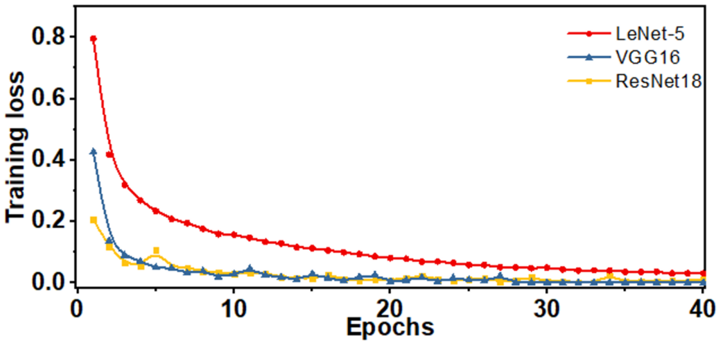

Figure S2: The training loss curves of three CNN models.

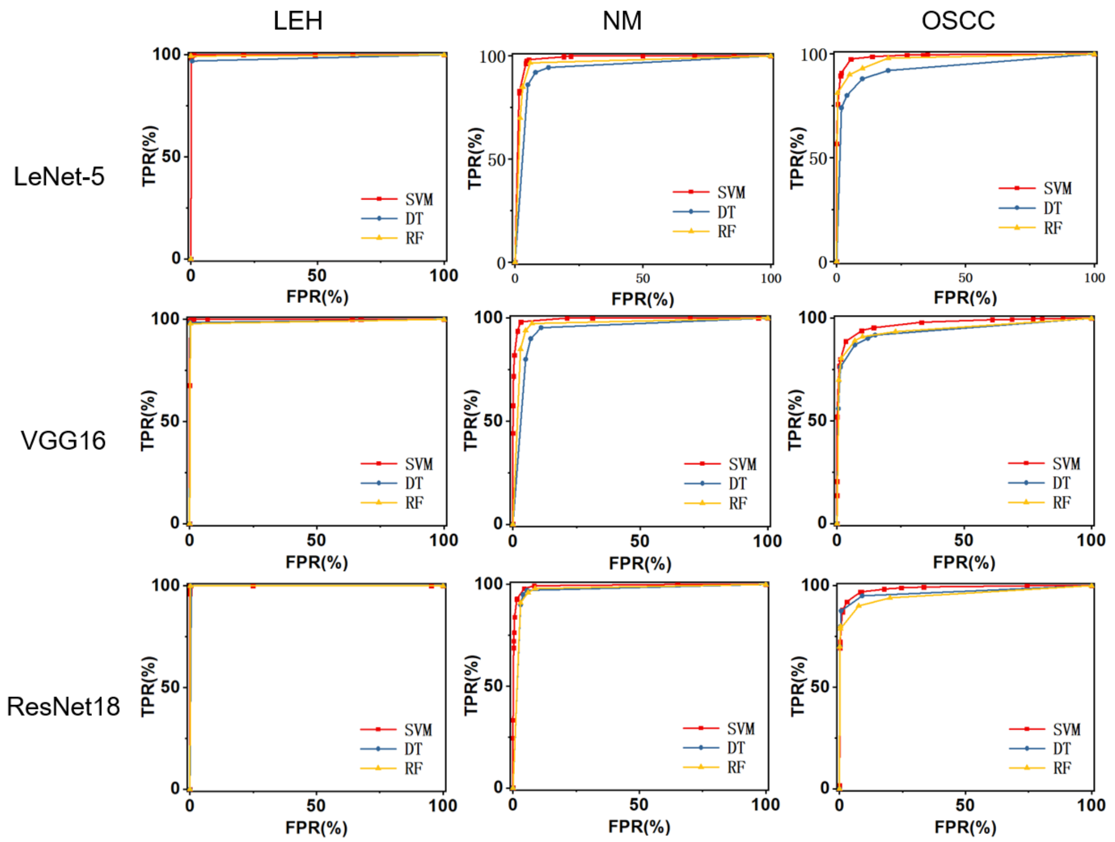

Figure S3: The ROC curves for SVM, DT, and RF as classifiers and LeNet-5, VGG16, and ResNet18 as feature extractors, respectively. ROC: receiver operating characteristic curve, FPR: false positive rate, TPR: true positive rate.

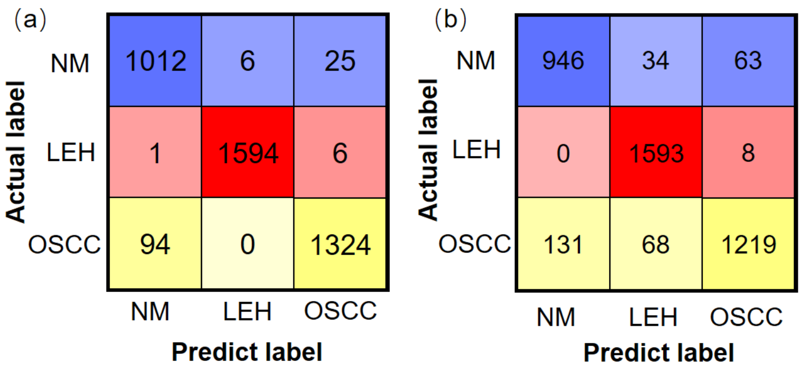

Figure S4: Confusion matrices of two strategies. (a) LeNet-5. (b) LeNet-5 with SVM as classifier and LeNet-5 as feature extractor.

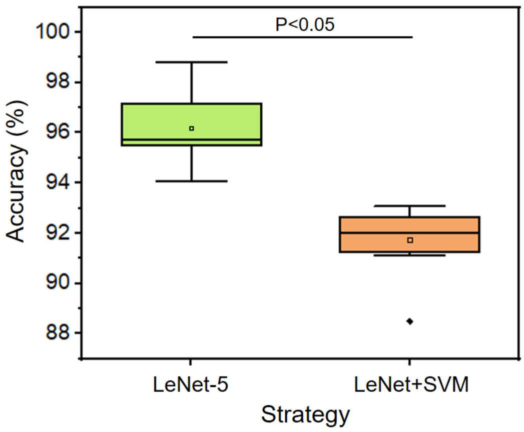

Figure S5: Statistical analysis of two strategies based on student's t test.
